# Pitfalls and perils of survival analysis under incorrect assumptions: the case of COVID-19 data

**DOI:** 10.7705/biomedica.5987

**Published:** 2021-10-15

**Authors:** Daniele Piovani, Georgios K. Nikolopoulos, Stefanos Bonovas

**Affiliations:** 1 Department of Biomedical Sciences, Humanitas University, Pieve Emanuele, Milan, Italy Department of Biomedical Sciences Humanitas University Milan Italy; 2 Medical School, University of Cyprus, Nicosia, Cyprus University of Cyprus Nicosia Cyprus; 3 IRCCS Humanitas Research Hospital, Rozzano, Milan, Italy IRCCS Humanitas Research Hospital Milan Italy

**Keywords:** Coronavirus infections, betacoronavirus, severe acute respiratory syndrome, survival analysis, data interpretation, statistical, infecciones por coronavirus, betacoronavirus, síndrome respiratorio agudo grave, análisis de supervivencia, interpretación estadística de datos

## Abstract

Non-parametric survival analysis has become a very popular statistical method in current medical research. However, resorting to survival analysis when its fundamental assumptions are not fulfilled can severely bias the results. Currently, hundreds of clinical studies are using survival methods to investigate factors potentially associated with the prognosis of coronavirus disease 2019 (COVID-19) and test new preventive and therapeutic strategies. In the pandemic era, it is more critical than ever to base decision-making on evidence and rely on solid statistical methods, but this is not always the case. Serious methodological errors have been identified in recent seminal studies about COVID-19: One reporting outcomes of patients treated with remdesivir and another one on the epidemiology, clinical course, and outcomes of critically ill patients.

High-quality evidence is essential to inform clinicians about optimal COVID-19 therapies and policymakers about the true effect of preventive measures aiming to tackle the pandemic. Though timely evidence is needed, we should encourage the appropriate application of survival analysis methods and careful peer-review to avoid publishing flawed results, which could affect decision-making.

In this paper, we recapitulate the basic assumptions underlying non-parametric survival analysis and frequent errors in its application and discuss how to handle data on COVID-19.

## Resumen

El análisis de supervivencia es un método estadístico muy popular en la investigación médica actual. Sin embargo, el recurrir al análisis de supervivencia cuando no se cumplen sus supuestos fundamentales puede sesgar gravemente los resultados. Actualmente, cientos de estudios clínicos están utilizando esta metodología para estudiar los factores potencialmente asociados con el pronóstico de la COVID-19 y probar nuevas estrategias preventivas y terapéuticas.

En la pandemia actual es más importante que nunca que las decisiones se basen en pruebas y en métodos estadísticos sólidos. Sin embargo, este no es siempre el caso. Se han detectado errores metodológicos graves en estudios seminales recientes sobre COVID-19: uno que informa los resultados de los pacientes tratados con remdesivir y otro sobre la epidemiología, el curso clínico y los resultados de los pacientes críticamente enfermos. La evidencia de calidad es esencial para informar a los médicos sobre las terapias óptimas contra la enfermedad y, a los legisladores, sobre el verdadero efecto de las medidas preventivas destinadas a abordar la pandemia. Aunque se necesitan pruebas oportunas, debemos fomentar la aplicación adecuada de los métodos de análisis de supervivencia y una cuidadosa revisión por pares para evitar la publicación de resultados defectuosos que pueden afectar la adopción de decisiones.

En este artículo, recapitulamos los supuestos básicos que subyacen al análisis de supervivencia y los errores frecuentes en su aplicación, y discutimos cómo manejar los datos sobre la COVID-19.

Non-parametric survival analyses are sophisticated statistical techniques that have become very popular in current medical research ^1^. These special methods are useful in studies with time-to-event data and can be applied to observational and experimental research alike. In such research studies, the outcome of interest can be “negative”, such as death or heart attack, or “positive”, such as hospital discharge, while an important feature of time-to- event data is that at the end of the follow-up time the outcome may not have occurred for all patients ^2^.

For the analysis of such data, we need to apply survival analysis methods; however, employing non-parametric survival analysis methods when their fundamental assumptions are not met can severely bias the results. In a recent study reporting outcomes in a cohort of patients hospitalized for severe COVID-19 and treated with remdesivir on a compassionate-use basis ^3^, the authors failed to appropriately consider the participants who died, thus overestimating the cumulative incidence of clinical improvement ^4^. Similar methodological errors have been identified in a seminal observational study on the epidemiology, clinical course, and outcomes of critically ill patients with laboratory-confirmed COVID-19 ^5^^,^^6^ and in influential randomized clinical trials conducted in other research fields ^7^^,^^8^.

Especially in the COVID-19 pandemic era, decision-making should be evidence-based and rely on solid statistical methods. Although the concepts presented in this paper are not novel, errors in handling survival data are still occurring frequently, even in leading medical journals ^3^^-^^8^. In this study, we recapitulate the basic assumptions underlying the most commonly used non-parametric methods for survival analysis in the medical field and discuss frequent errors in their application including scenarios of competing risks and a certain fallacy that may occur when studying COVID-19 or other acute infections.

## Time-to-event data and censoring

In survival analysis, we analyze the numbers of participants who suffered the event of interest (i.e., a dichotomous variable of event status) and the times at which the events have occurred (i.e., a continuous variable, which reflects the time until a patient has the event of interest) ^2^.

Participants who have not experienced the event of interest before the end of the follow-up period are defined as “censored”, i.e., their observation period has ended before event occurrence. This is also called administrative censoring. In these participants, the probability to experience the event of interest after the date of censoring is unknown ^9^. Censoring may also happen when patients are lost to follow-up, have withdrawn from the study, or when other events have prevented further follow-up (i.e., competing events). In each of these cases, we have incomplete follow-up information ^2^.

Prior to any analysis, the dataset should be adequately compiled with a continuous variable reporting the time of follow-up and, typically, a categorical variable taking value zero if the follow-up has been censored, one if the event of interest has occurred, and other values if one or more (competing) events have prevented the observation of the event of interest. In this paper, we will refer to “right-censoring”, as this is the most frequent scenario encountered in the medical literature, but other types of censoring also exist.

## Basic assumptions of non-parametric survival methods

Although alternative methods exist, estimating the survival probability of a group of patients is traditionally performed through the Kaplan-Meier method ^10^^,^^11^. This step function takes into account censoring and computes the proportion of patients surviving (i.e., not experiencing the event of interest) at each timepoint an event occurs until the end of the follow-up period ^10^^,^^11^. To correctly apply the Kaplan-Meier method and avoid flawed results, censoring should be “non-informative” ^9^^,^^11^, in other words, patients who are censored should have the same future risk for the occurrence of the event of interest, conditional on exposure and covariates, as those who continue to be followed. This is referred to as “assumption of independent censoring”, which is often referred to as non-informative censoring, though the latter has a slightly different technical definition. If the risks are different, the censoring assumption is violated: Censoring the former group of patients can introduce bias when estimating the survival probability through the Kaplan-Meier method ^11^.

Often the aim of epidemiological studies is the formal comparison of the survival prospects between two or more groups of patients (e.g., receiving different treatments or having different baseline characteristics). The most popular survival analysis method for this purpose in medicine is the Cox proportional hazards model ^12^. This regression method is widely used for investigating the association between patients' survival time and one or more categorical or continuous variables. A fundamental assumption is that the ratio of hazards of any two individuals (hazard is the instantaneous rate of event occurrence conditional on survival) should remain roughly constant at all the timepoints since baseline. This is often referred to as “proportional hazards assumption”; it is the baseline assumption for applying the log-rank test and the proportional Cox regression method ^12^.

Under this assumption, comparing the Kaplan-Meier curves of two subgroups of patients having different survival prospects should show approximately parallel functions while an important deviation from proportionality would determine a visible relative change in slope along time. As a consequence, when the proportionality assumption is not met, the estimated hazard ratios depend largely on the follow-up time ^13^. Proportionality can be checked through tests or graph-based methods based on Schoenfeld residuals. Previous studies on randomized controlled trials have shown that using a Cox regression model when this assumption is not met can systematically inflate the magnitude of the effect associated with a given treatment ^14^. Other appropriate methods to analyze survival data in case of non-proportional hazards also exist, such as parametric survival methods (e.g., Royston-Parmar model), which do not rely on this assumption ^15^.

## Competing risks in survival analysis

On several occasions, the chance to observe the outcome of interest can be altered or prevented by the occurrence of a competing event ^16^. For instance, in a study investigating breast cancer recurrence, we might want to know whether the recurrence rate differs between two or more treatment groups. Death from any cause prior to breast cancer relapse (e.g., from a heart attack or stroke, or even from a traffic accident) is a “competing event” whose occurrence precludes the primary outcome of interest. Accordingly, we call the probability of these events “competing risks” because the probability of each competing event is regulated by the other competing events.

A similar competing risks scenario in COVID-19 could be observed in a study evaluating the effect of a certain vaccine on COVID-19-associated mortality. Especially very old people who were given priority in vaccination may die during the follow up from another cause without even being infected. Death associated with COVID-19 pneumonia and death from another cause are not independent events. When death answers to another cause, patient follow-up is interrupted and the probability of dying due to consequences of COVID-19 becomes zero. However, death is not a random event; patients who die from another cause might not have the same probability of dying from COVID-19 pneumonia as those who continue to be followed (e.g., patients who die are generally older and with more comorbidities). Hence, censoring of patients who died for any other cause because the follow-up stops violates the independent censoring assumption (i.e., the event “death from another cause” is informative), thus biasing the cumulative incidence of death associated with COVID-19 pneumonia. On the contrary, if patients dying from other causes are not censored, we assume that it is possible to die due to the consequences of COVID-19 pneumonia after having already died - a completely unrealistic scenario.

## How do we deal with competing risks in survival analysis?

The application of the Kaplan-Meier method is not appropriate in such cases; it can lead to flawed results (i.e., to an overestimation of the cumulative incidence of the event of interest) ^17^^,^^18^. Instead, estimating the cumulative incidence function through alternative methods is the correct methodological approach ^19^^,^^20^. The cumulative incidence function is a product of two probabilities: In this example, the first term would be the probability that death associated with COVID-19 happens up to time t, the second term would be the probability that this event is COVID-19-associated death (and not the competing event, i.e., death from another cause). Cumulative incidence function curves can be calculated and plotted for both COVID-19-associated death and death from another cause.

The cumulative incidence function can be seen also as the probability of observing the event of primary interest up to a certain timepoint ^16^. In other words, this method allows estimating the hazard of event occurrence while taking into account that one or more events could compete with the primary outcome of interest. In a scenario with no competing events, the Kaplan-Meier method and the cumulative incidence function approach would give exactly the same result.

A second important problem of using the Kaplan-Meier method in the presence of competing risks and informative censoring occurs when the aim of the study is to formally compare the survival probability between two or more groups. For example, in a randomized controlled trial, this error might bias not only the overall survival probability but also the absolute difference in risk between the treatment and comparator groups, thus distorting the number needed to treat and the number needed to harm ^21^. In observational studies, this could potentially bias the hazard ratio estimated through Cox proportional-hazards regression models.

For example, let's assume that in our previous example of COVID-19 vaccination, the aim was instead to formally compare vaccine A and vaccine B in terms of COVID-19-associated mortality. As already stated, censoring patients who die from another cause and using the Kaplan-Meier approach would overestimate the cumulative incidence of the event under scrutiny (i.e., COVID-19-associated death) in both patient groups. This could either inflate or deflate the absolute risk difference between groups depending on multiple factors, including the frequency and timing of competing events across comparison groups, and the actual magnitude of the risk difference ^21^. Additionally, if the study design was observational, unmeasured, or residual confounding could yield an additional distortion of the hazard ratio. This would occur if one or more characteristics of the patients are differentially associated with the probability of the event of interest (COVID- 19-associated death) and of the competing event (death from another cause). The direction of this distortion is not easy to predict. The solution for this issue in the current example is to avoid using the Kaplan-Meier method and Cox proportional-hazards regression and, instead, apply the Fine and Gray model, which appropriately considers the two sub-distribution cumulative incidence functions (i.e., of relapse and death) in the two groups of patients ^22^ or, alternatively, the cause-specific cumulative incidence function. In these studies, confounding should be addressed at the design stage by collecting well-known confounders and at the analysis stage by stratification matching or including confounders in the multivariable regression model. Given the observational nature of the data, residual confounding associated with unmeasured or unknown confounders cannot generally be excluded.

Competing-risks methods are being increasingly applied in the analysis of cause-of-death data to obtain real-world probabilities of death broken down by specific causes. This information is crucial for informing patients about the risks they face in certain conditions and for making evidence-based decisions about optimal therapies and the best healthcare resource allocation.

In literature, it has been proposed that competing-risks methods must be considered in the analysis when the percentage of patients having experienced the competing event is higher than that of patients having experienced the event of interest ^23^, or when the absolute percentage of patients having experienced the competing event is higher than 10 percent ^(16)^. Although competing-risks regression methods based on the cumulative incidence function, such as the Fine and Grey model, have been known for two decades ^22^, failing to appropriately account for competing risks in statistical analysis is not uncommon ^7^^,^^8^^,^^14^^,^^21^. However, is it always necessary to apply competing-risks regression in the analysis of time-to-event data with competing events?

## The case of COVID-19 and other acute infections

Herein, we present a scenario in which the Kaplan-Meier method can be applied to correctly handle a special case of a competing event. We refer to acute infections requiring hospitalization such as for example, COVID-19 pneumonia. This example also applies to the COVID-19 studies previously mentioned ^3^^-^^6^.

We hypothesize a clinical study involving patients admitted to hospital for COVID-19 pneumonia in which the primary outcome is “mechanical ventilation or in-hospital death” within 28 days after admission. How should we analyze the data for patients who have been discharged alive?

A discharge event prevents the observation of the study outcome by interrupting the follow-up. Since the discharge is not a random event, censoring these patients (on the date of their discharge) would constitute informative censoring; this would lead to inflated results by overestimating the risk of “mechanical ventilation or in-hospital death” among patients admitted to the hospital for COVID-19 pneumonia ^6^.

An option to resolve the issue is to use the cumulative incidence function methodology. However, is it really necessary to use competing-risks analysis in this case? Let's reconsider the previous example regarding the COVID-19 vaccine in which death for any cause was the competing event for COVID-19- associated death. These two events are both “negative” and the probability of dying from another cause is likely not independent of the study outcome. By using the cumulative incidence function, we quantitatively assess the extent of this association through the estimation of two distinct sub-distribution cumulative hazard functions (one for the event of primary interest and one for the competing event), which correctly take into account the competitive nature of both events ^16^.

In the current example of critically ill adults with laboratory-confirmed COVID-19 admitted to hospitals in New York City ^5^, discharge (competing event) is the “opposite” of the primary outcome of interest (mechanical ventilation or in-hospital death) and the dependence between the two events is very clear. The probability of outcome occurrence at and after discharge is zero because the patient has recovered. If we could follow this patient after discharge, she/he would be event-free until the end of the observation period (28 days after admission to the hospital). This is not a proper competing- risks scenario because, in the short 28-day timeframe, COVID-19 can be considered an acute illness, and, once discharged, a patient will not relapse. For these reasons, in this example, a correct approach is to consider the patients discharged as censored at the end of the study follow-up (day 28) and use the Kaplan-Meier method ^6^.

The same approach also applies to the exactly specular scenario: Having “clinical improvement” as the primary outcome of interest and in-hospital death for COVID-19 as the competing event ^3^^,^^4^.

Recently, this simple method has been nicely applied by authors of randomized controlled trials on remdesivir ^25^^,^^26^, dexamethasone ^27^, hydroxychloroquine ^28^, and lopinavir/ritonavir ^29^ for the treatment of COVID-19 patients.

## Discussion and conclusion

Nowadays, there are appropriate statistical methods and powerful software to correctly analyze time-to-event data in the presence or absence of competing risks ^9^^-^^12^^,^^17^^,^^19^^,^^21^^,^^22^. The choice of the approach used should be driven by the nature of the data and the scientific question itself. In general, the Kaplan-Meier method is not appropriate in circumstances where there are competing risks. This is particularly true if censoring is applied to competing events occurring with high frequency. Failure to account correctly for competing events can lead to overestimating the outcome occurrence and to flawed estimates of effect in clinical studies examining the effect of covariates (e.g., treatments or patient characteristics) on the incidence of the outcome of interest. When the outcome of the study is clinical improvement, this may lead to overestimating the beneficial effect of experimental drug treatment ^3^^,^^4^, thus giving credits to potentially ineffective drugs and leading to a waste of time and resources.

Survival analysis is increasingly employed in the current medical literature; however, authors often do not explain the method used to deal with censoring. Given the frequent occurrence of situations with competing risks and the biases resulting from incorrectly analyzed time-to-event (survival) data, manuscript reviewers should encourage researchers to use optimal statistical approaches. As we have shown, even the most influential journals in medicine are not immune to such issues ^3^^-^^6^. Readers of the medical literature should be aware of these potential problems and should look for information on censoring methods. To increase transparency, we advise that the method of censoring should be clearly reported in the methods sections and persons at risk should be always declared below the graphs that are plotting the survival probability or the cumulative incidence curves.

In the pandemic era, hundreds of observational studies and experimental trials are investigating factors associated with the prognosis of COVID-19 patients and testing new treatments ^30^^,^^31^. High-quality evidence is essential to inform clinicians about optimal COVID-19 therapies and policymakers about the true effect of preventive measures aiming to tackle the pandemic. Raising false expectations regarding treatments because of errors in the statistical methods is unacceptable and should be avoided at all costs, especially in these difficult times. Though timely evidence is needed, we should encourage the appropriate application of survival analysis methods and careful peerreview to avoid publishing flawed results, which could affect decision-making.
